# The Neuroprotective Effects of Exercise on Cognitive Decline: A Preventive Approach to Alzheimer Disease

**DOI:** 10.7759/cureus.6958

**Published:** 2020-02-11

**Authors:** Muhammad Humayoun Rashid, Muhammad Farhan Zahid, Sarmad Zain, Ahmad Kabir, Sibt Ul Hassan

**Affiliations:** 1 Neurology, Bakhtawar Amin Medical and Dental College, Multan, PAK; 2 Anatomy, Bakhtawar Amin Medical and Dental College, Multan, PAK; 3 Internal Medicine, Nishtar Hospital, Nishtar Medical University, Multan, PAK; 4 Pathology, Bakhtawar Amin Medical and Dental College, Multan, PAK; 5 Internal Medicine, Bakhtawar Amin Memorial Hospital, Multan, PAK

**Keywords:** exercise, alzheimer disease, cognitive decline, review article, neurofibrillary tangles, amyloid plaques, dementia, neuroinflammation, neurogenesis, angiogenesis

## Abstract

Alzheimer's disease (AD) is a progressive disorder that causes brain cells to slowly degenerate and die. This leads to a continuous decline in thinking, behavioral and social skills that disrupts a person's ability to function independently. AD is the most common cause of dementia globally. Neuroinflammation caused by intracellular neurofibrillary tangles and extracellular amyloid deposits leads to atrophy of brain cells especially the hippocampus, which is associated with memory formation. This atrophy leads to dementia and cognitive decline. Among the many preventive factors being studied, exercise is thought to play a vital role in not only preventing the pre-clinical stage of AD but also slowing the clinical progression of AD. It is also deployed as a treatment option for late-stage AD along with pharmacological treatment options. Various studies and clinical trials in both human and animal models are of the opinion that exercise slows the onset and progression of cognitive decline in AD patients. Some studies suggest that this effect is due to a decrease in neurofibrillary tangles and amyloid deposits in brain parenchyma. Others suggest that exercise causes an increase in angiogenesis, neurogenesis, and synaptogenesis mainly due to an increase in blood flow, brain-derived neurotrophic factor (BDNF), insulin-like growth factor 1 (IGF-1), hormones, and second messengers.

## Introduction and background

Physical exercise is crucial for maintaining general body health. It helps to maintain body weight and reduces stress level. People who exercise regularly are less likely to smoke cigarettes or overeat. More than that, exercise directly targets primary aspects of health such as heart functioning, cholesterol, triglycerides, blood pressure and also a brain. It is known that regular exercise, which leads to enhanced maximal oxygen uptake, increases the mean lifespan of laboratory animals and humans [1-3]. Exercise is known to impact nearly every system of the body. Benefits of regular exercise include improved cardiovascular health, greater bone mineral density (BMD), and decreased risk of cancer, stroke, diabetes, and cognitive impairment, yet many people still choose not to participate.

The form of exercise most often connected with decreasing heart diseases and increasing neuronal life span is aerobic exercise [4]. Aerobic exercise can decrease the risk of heart disease by 20 to 60 percent, depending on the exertion level, duration, and frequency. Apart from physical health, people who exercise regularly tend to do so because it gives them an enormous sense of well-being. They feel more energetic throughout the day, sleep better at night, have sharper memories and feel more relaxed and positive about themselves and their lives. It is also powerful medicine for many common mental health challenges.

Regular exercise can have a profoundly positive impact on depression, anxiety, attention-deficit/hyperactivity disorder (ADHD), and more [5]. It also relieves stress, improves memory, helps to sleep better, and boosts overall mood. And it does not require being a fitness fanatic to reap the benefits. Researches indicate that a modest amount of exercise can make a difference. Regardless of age or fitness level, one can learn to use exercise as a powerful tool to feel better. Physical inactivity is one of the strongest risk factors for dementia, Parkinson's disease and Alzheimer's disease [6-8]. 

The purpose of this review is to highlight the neuroprotective impacts of exercise on Alzheimer's disease so that the morbidity and mortality associated with this disease can be reduced, as prevention is better than cure. Amyloid-beta deposits, neurofibrillary tangles, neurotrophic factors, oxidative stress, and overall blood flow to the brain play a very crucial role in developing Alzheimer's disease. Many of the researchers are of the view that exercise protects neuronal tissue from these factors mentioned above. Exercise is found to increase the life span of neurons in animal studies.

Alzheimer's disease and pathophysiology

**Figure 1:**
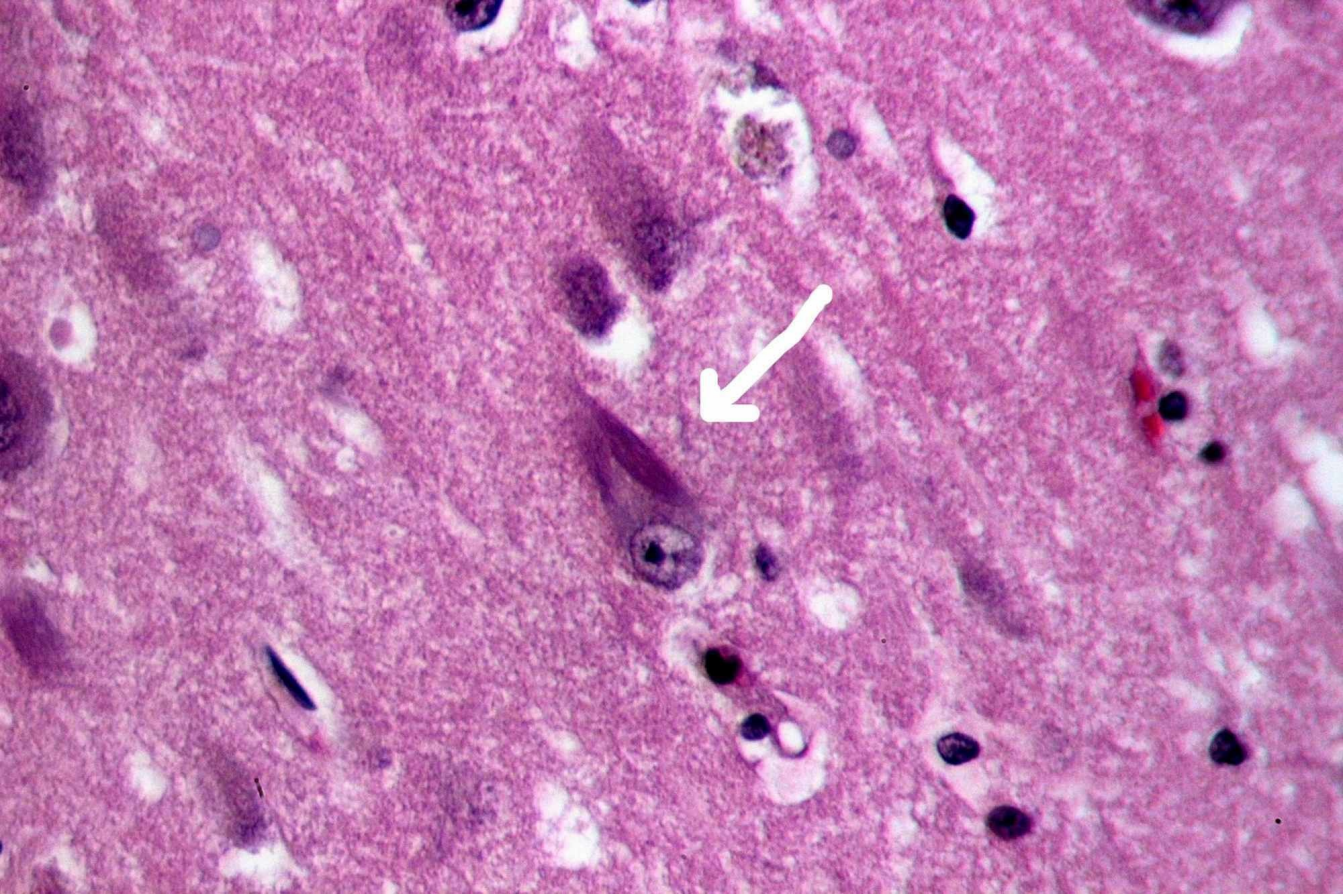
Neurofibrillary tangle (white arrowhead) Recreated under creative commons license [14].

**Figure 2:**
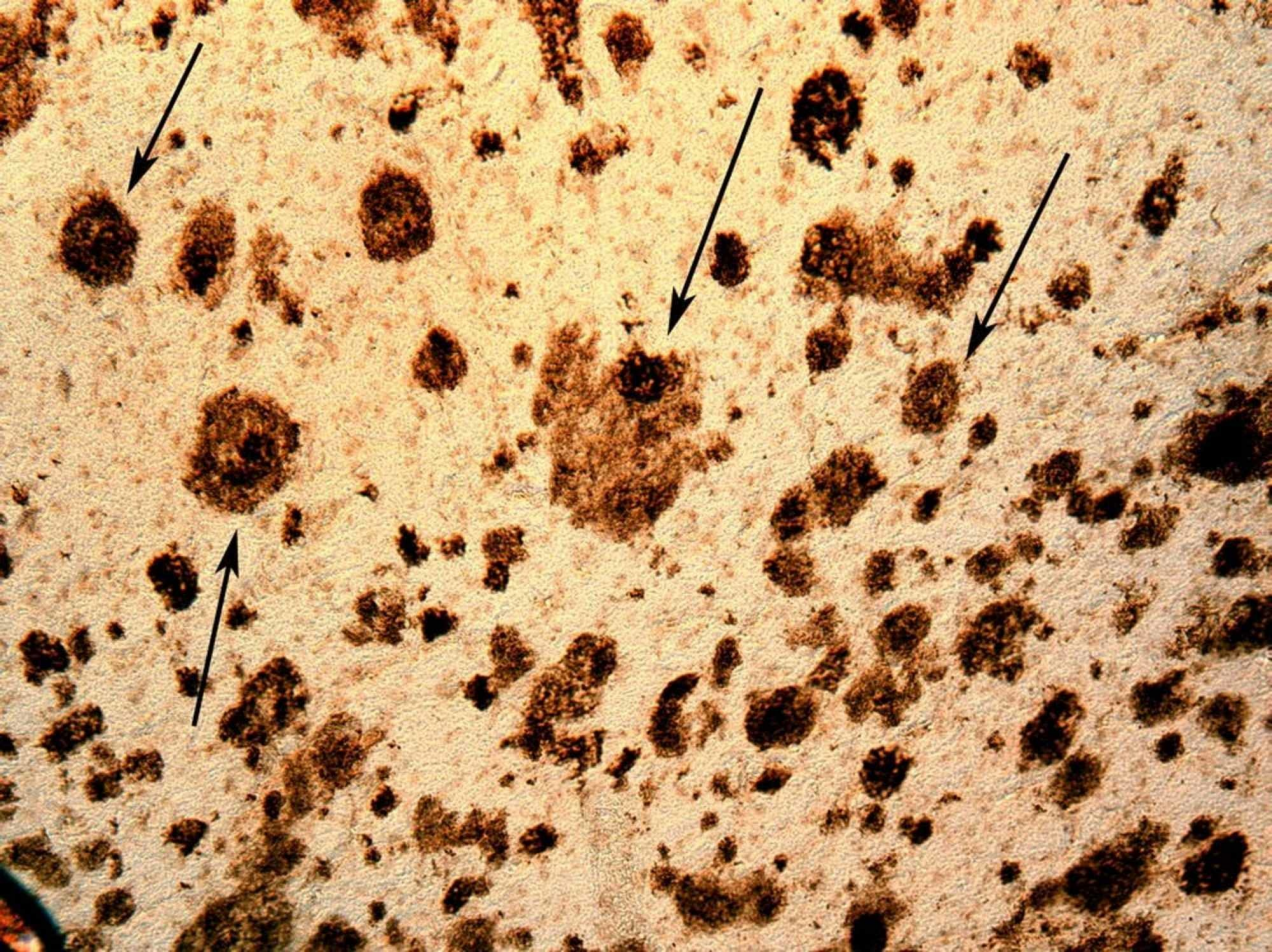
Amyloid plaques (black arrowheads) Recreated under creative commons license [14].

Alzheimer's disease (AD) is classically defined as a dual clinicopathological entity [9]. It is a progressive disorder that causes brain cells to waste away (degenerate) and die. It is the most common cause of dementia - a continuous decline in thinking, behavioral and social skills that disrupts a person's ability to function independently. Recently recognized as the prodromal stage of AD, mild cognitive impairment (MCI) represents a transitional period between normal aging and AD [10, 11]. MCI is determined to be the initial stage of AD and hence is frequently used as the initial clinical tool to assess AD, especially the MCI associated with amnesia. Amnestic MCI has a transition rate of about 10 - 15% per year [12]. Alzheimer’s disease is progressive dementia with loss of neurons and the presence of two main microscopic neuropathological hallmarks: extracellular amyloid plaques and intracellular neurofibrillary tangles (see Figures 1 and 2). Plaques develop in the hippocampus, a structure deep in the brain that helps to encode memories, and in other areas of the cerebral cortex that are involved in thinking and making decisions [13]. 

Amyloid precursor protein (APP) is part of a superfamily of transmembrane and secreted proteins. It appears to have a number of roles, including regulation of hemostasis and mediation of neuroprotection. APP also has potentially important metal and heparin‐binding properties, and the current challenge is to synthesize all these varied activities into a coherent view of its function. Cleavage of amyloid precursor protein by β‐ and γ‐secretases results in the generation of the Aβ (βA4) peptide, whereas α‐secretase cleaves within the Aβ sequence and prevents formation from APP. Recent findings indicate that the site of γ‐secretase cleavage is critical to the development of amyloid deposits [15]. Plaques are dense insoluble substances deposited outside the cell whereas tangles are hyperphosphorylated tau proteins that are deposited intracellularly. The most common location of these deposits is the temporal lobe. Both Aβ and intracellular neurofibrillary tangles favor the excessive accumulation of reactive oxygen species (ROS) due to mitochondrial dysfunction which increases oxidative stress. This leads to increased levels of lipid and protein peroxidation causing neurodegeneration and cerebral amyloid angiopathy [16]. Small vessel disease (SVD) involving arteries, arterioles and capillaries also play a role in AD progression due to micro infarctions in various brain regions. SVD and AD are both associated with many morbidities in later stages of life.

**Figure 3:**
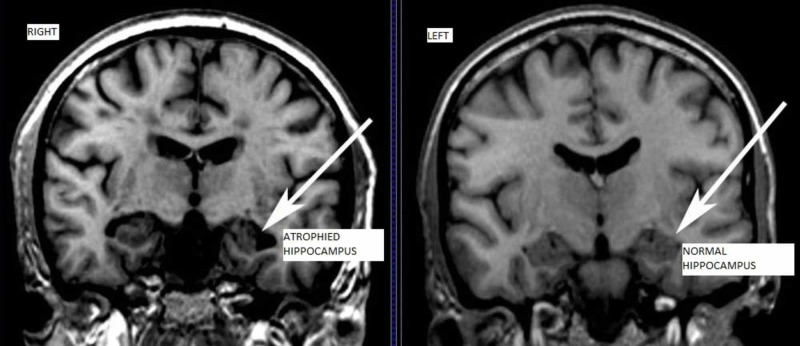
MRI showing atrophy in hippocampus indicated with arrows Arrowhead in the right image shows atrophied hippocampus in AD patient whereas the arrowhead in the left image shows normal hippocampus. AD - Alzheimer's disease Recreated under creative commons license [14].

AD progresses through pre-dementia, early, moderate and advanced disease. The early signs of the disease may be forgetting recent events or conversations. Problems with planning, attentiveness, executive functions, and semantic memory may occur. As the disease progresses features such as wandering, irritability, lack of insight about the disease and anosognosia may develop [17]. A person with Alzheimer's disease will develop severe memory impairment and lose the ability to carry out everyday tasks. During advanced disease, a person may lose the ability to talk and become completely dependent on the caregivers. Exhaustion and extreme apathy are common symptoms [17, 18]. AD can be both hereditary and sporadic. Hereditary AD is often associated with mutations in presenilin 1, presenilin 2 or amyloid precursor protein [19]. Sporadic AD is associated with apolipoprotein E (APOE) and is under environmental influences [20]. APP gene is present on chromosome 21 and hence people present with Down syndrome present with AD in the early 40s [14]. Figure 3 shows the gross changes that appear in the brains of AD patients.

Exercise and brain health

Exercise is thought to have both direct and indirect effects on brain health. Among the indirect effects, it includes reducing the incidence of chronic diseases such as coronary heart disease and some lung diseases that impact neurocognitive functions [21-24]. Adequate pumping and proper oxygenation of blood both complement each other and supplies a healthy environment for visceral growth. The brain also depends on both of these factors for healthy growth and proper functioning. The direct effects of exercise on brain health are thought to be at two levels, the molecular and the supramolecular levels. Both of these levels are currently being researched and still debatable. The supramolecular level includes angiogenesis, synaptogenesis, and neurogenesis which are then controlled at the molecular level by the brain-derived neurotrophic factor (BDNF), insulin-like growth factor 1 (IGF-1), hormones and second messengers [25].

The blood supply of the brain is modifiable and depends on various factors. One of these is a physical activity that increases the vascular supply of the brain, which ultimately makes the brain grow healthy [26]. Various animal and human studies have shown a transient increase in blood flow to the brain during skilled motor tasks and also for a brief period after cessation of physical activity [27-28]. It has been well understood that neurogenesis continues to occur in different parts of the brain in various situations such as after a stroke, ischemia, or other injuries. The fact that physical activity can also promote neurogenesis is under consideration and still being researched in various animal and human models. Factors such as hormones, neurotransmitters, growth factors, and drugs can stimulate neurogenesis [29-30]. 

Apart from angiogenesis and neurogenesis, synaptogenesis is also affected by exercise. The modification of pre- and postsynaptic clefts and the increase in the synthesis of synaptic vesicles are associated with a healthier brain. The effects of physical activity on synaptogenesis were studied by Molteni and his colleagues, Farmer J, Zhao X, and their coworkers and Figurov A, Pozzo-Miller LD and their co-researchers [31-33]. All of them are of the opinion that continuous moderate exercise increases the expression of genes concerned with synaptogenesis, and this is associated with an increase in cognitive functions of the brain.

Exercise, neurofibrillary tangles and amyloid plaques

This topic is highly being researched in various parts of the world and the reason being is the lack of clarity about the association between them. Neurofibrillary tangles are insoluble hyperphosphorylated tau proteins that are deposited intracellularly and cause neuroinflammation. This neuroinflammation leads to the progression of AD. The more the neurofibrillary tangles, the worse the prognosis of AD is. Neurofibrillary tangles are seen on the histopathology of autopsy specimens of people with AD. Similarly, extracellular amyloid deposits are also found to be associated with neuroinflammation and AD progression.

New researches show that physical exercise can “clean up” the hostile environments in the brains of Alzheimer’s mice, allowing new nerve cells in the hippocampus, the brain structure involved in memory and learning, to enable cognitive improvements such as learning and memory. Leem YH and his colleagues [34] studied that chronic endurance exercise can reduce the hyperphosphorylation of tau proteins in the aged transgenic mouse model. Chronic endurance exercise in transgenic mice increased the expression of Cu/Zn‐superoxide dismutase (SOD) and catalase, and also their enzymatic activities in the brain. In parallel, a chronic exercise in transgenic mice up‐regulated the expression of phospho‐PKCα, phospho‐AKT, and phospho‐PI3K, and down‐regulated the expressions of phospho‐PKA, phosphor‐p38, phospho‐JNK, and phospho‐ERK. Moreover, chronic exercise up‐regulated both cytosolic and nuclear levels of β‐catenin, and the expression of T‐cell factor‐4 (Tcf‐4) and cyclin D1 in the brain. These changes reduce the neuroinflammation caused by neurofibrillary tangles in Alzheimer's disease. Ohia-Nwoko O studied that exercise improved general locomotor and exploratory activity and resulted in significant reductions in full-length and hyperphosphorylated tau in the spinal cord and hippocampus as well as a reduction in sarkosyl-insoluble AT8-tau in the spinal cord [35].

Many studies indicate that continuous exercise is also associated with a decrease in amyloid deposits in the brain. Azimi M, Gharakhanlou R studied that moderate treadmill exercise ameliorated the Aβ1−42-induced spatial learning and memory deficit, which was accompanied by restored adenosine monophosphate-activated protein kinase (AMPK) activity and PGC-1α/FNDC5/BDNF levels [36]. Li B and Liang F studied the effects of two exercise modes on cognitive function and mitochondrial dynamics in APP/PS1 transgenic mice [37]. The results showed that 12-week high-intensity interval training (HIIT) and moderate-intensity continuous training (MICT) could improve exploratory behavior, spatial learning and memory ability of APP/PS1 transgenic mice. Both HIIT and MICT interventions significantly alleviated the hippocampal β-Amyloid (Aβ) burden and mitochondrial fragmentation and improved mitochondrial morphology in the hippocampus.

Another study was associated with the fact that short term treadmill exercise increases the insoluble hyperphosphorylated tau proteins in the brain and hence increase the neurofibrillary tangles. Elahi M and his co-workers found out that short term treadmill exercise increases the oxidative stress in the brain which then increases hyperphosphorylation of tau proteins and cause neuroinflammation [38]. Hence the topic is still being studied and various trials are currently in progress.

## Review

The biological mechanisms by which cognition is enhanced through physical training remain to be completely elucidated, although the number of studies that have tried to identify these mechanisms has increased in the last 10 years. For the most part, the studies that support the notion that physical exercise has an impact on brain functions have focused on direct biological effects of exercise using both animal and human models. While going through various clinical trials done by different researches in various setting it was observed that the impact of exercise on slowing AD progression is quite significant.

Buchman AS and his colleagues found that there is a strong association between exercise and the risk for developing AD [39]. People with low activity were more likely to develop AD compared to those with high rates of activity (HR=0.477; 95% CI: 0.273-0.832). The risk of developing AD was two times higher. Rolland Y and his colleagues did a randomized controlled trial of one year in five different nursing homes and found out that one-hour exercise twice a week is associated with a slower progression of Alzheimer’s dementia than routine medical care [40].

Kim DY et al. observed that treadmill exercise can reduce Alzheimer associated memory loss in Streptozotocin-induced diabetic rats [41]. Adlard et al. studied the impact of exercise on dementia progression in AD [42]. They made transgenic mice with CRND gene mutation at position 8 (TgCRND8) and observed the extracellular deposits of amyloid plaques in different brain regions. They studied that five months of voluntary exercise resulted in decreased amyloid-beta deposits in the frontal cortex (38%; p=0.018), the cortex at the level of the hippocampus (53%; p=0.0003), and the hippocampus (40%; p=0.06). Adlard and his colleagues concluded that it was due to decreased fragmentation of amyloid precursor proteins (APP). Akbaraly et al. studied the impact of leisure activities in people older than 65 years. A three-city cohort study in Dijon and Montpellier (France) in 1999-2001 was done. They found out that stimulating leisure activities were found to be associated significantly with a reduced risk of dementia (n=161, HR=0.49, 95% CI: 0.31; 0.79) and Alzheimer's disease (n=105, HR=0.39, 95% CI: 0.21; 0.71) over the four-year follow-up [43].

Etgen et al. also did a cohort analysis of the impact of moderate to high physical activity on cognitive impairment [44]. In Southern Bavaria, Germany, 3,903 participants older than 55 years were enrolled between 2001 and 2003 and followed up for two years. They found out that compared to participants without physical activity, those with moderate to high physical activity had a reduced risk of incident of cognitive impairment (OR: 0.57; 95% CI: 0.37-0.87, p=0.01; and OR: 0.54; 95% CI: 0.35-0.83, p=0.005 respectively). 

Vreugdenhil et al. performed four months of a randomized controlled trial to see the effect of exercise on the functional ability of AD patients [45]. They conducted a home-based exercise program and found out that at four‐months follow‐up, patients who exercised, compared with controls, had improved cognition (increased Mini-Mental State Examination scores by 2.6 points, p<0.001), better mobility (2.9 seconds faster on Timed Up and Go, p=0.004) and increased Instrumental Activities of Daily Living scores by 1.6 (p=0.007). 

Morris et al. recently studied the role of physical exercise as a therapeutic strategy in AD [46]. They carried out a 26-week randomized controlled trial and compared the effect of 150m aerobic exercise vs non-aerobic stretching. Aerobic exercise was associated with a modest gain in functional ability (Disability Assessment for Dementia) compared to individuals in the stretching and toning (ST) group (X2=8.2, p=0.02). Similarly, many of the recent trials done by various researchers such as Borges-Machado F, Ribeiro Ó, Hackney ME, McCullough LE, Ptomey LT, Vidoni ED, Lu X, Moeini M all concluded that exercise is a very vital component in slowing the progression of cognitive decline in AD patients [47-50]. A review of the various clinical trials is listed in Table 1. 

**Table 1:** Summary of various clinical trials on neuroprotective impacts of exercise over the years AD - Alzheimer’s dementia; ADL - activities of daily living; GSK-3B - glycogen synthase kinase 3-beta; Wnt3 - wingless related integration site 3; TgCRND8 - transgenic mice with CRND gene mutation at position 8; ST - stretching and toning; HR - hazard ratio; CI - confidence interval; OD - odds ratio

Author(s), year	Country	Trials	Findings	Summary
Buchman AS, Boyle PA [39] 2012	USA (Chicago)	A prospective four-year observational cohort study	People with low activity were more likely to develop AD compared to those with high rates of activity (HR=0.477; 95% confidence interval 0.273-0.832).	A higher level of total daily physical activity is associated with a reduced risk of AD.
Rolland Y, Pillard F [40] 2007	France	Randomized, controlled trials in five nursing homes for 12 months	Baseline score for exercise program patients showed a slower decline than in patients receiving routine medical care (12‐month mean treatment differences: ADL=0.39, p=0.002).	A simple exercise program, one hour twice a week, led to a significantly slower decline in ADL score in patients with AD living in a nursing home than routine medical care.
Kim DY, Jung SY [41] 2016	Korea	A clinical trial of treadmill exercise in rats for 12 weeks	Treadmill exercise increased Wnt3 expression and suppressed GSK-3β expression in diabetic rats.	Treadmill exercise can reduce Alzheimer's associated memory loss in streptozotocin-induced diabetic rats.
Adlard PA, Perreau VM [42] 2005	USA (California)	A clinical trial of five months voluntary exercise in TgCRND8 mice	Resulted in decrease in extracellular amyloid-B (AB) plaques in the frontal cortex (38%; p= 0.018), the cortex at the level of the hippocampus (53%; p=0.0003), and the hippocampus (40%; p=0.06).	Exercise is a simple behavioral intervention sufficient to inhibit the normal progression of AD.
Akbaraly TN, Portet F [43] 2009	France	A three-year cohort study on 5,698 dementia-free participants aged 65 and over	Leisure activities reduces risk of dementia (n=161, HR=0.49; 95% CI: 0.31; 0.79) and Alzheimer's disease (n=105, HR=0.39, 95% CI: 0.21; 0.71).	Cognitively stimulating leisure activities may delay the onset of dementia in community-dwelling elders.
Thorleif Etgen, MD [44] 2010	Germany	Cohort analysis of 3,903 participants older than 55 years.	Compared to participants without physical activity, those with moderate to high physical activity had a reduced risk of incident of cognitive impairment (OR: 0.57; 95% CI: 0.37-0.87, p=0.01; OR: 0.54; 95% CI: 0.35-0.83, p=0.005 respectively).	Moderate to high physical activity reduces cognitive impairment.
Morris JK, Vidoni MD [46] 2017	Australia	A 26-week randomized controlled trial	Aerobic exercise was associated with a modest gain in functional ability (Disability Assessment for Dementia) compared to individuals in the ST group (X2=8.2, p=0.02).	Aerobic exercise in early AD is associated with benefits in functional ability.
Borges-Machado F [47] 2019	Portugal	Quasi-experimental, nonrandomized study on 13 community-dwelling individuals diagnosed with probable AD	Significant beneficial effect on cardiorespiratory fitness (p=0.028), body muscle strength, agility (p=0.018), and ability to perform daily activities (p=0.018).	Findings suggest a potentially positive effect on mitigating cognitive decline and in positively influencing the quality of life.
Hackney ME, McCullough LE [48] 2019	USA	12-week, randomized, placebo-controlled Phase 1 clinical trial		The study showed the healthy impacts of exercise on AD.

## Conclusions

Both old and current studies indicate that exercise plays a vital role in reducing the incidence and slowing the progression of Alzheimer's disease. It is not clear if the relationship is dose-dependent or not, but it would appear that higher levels of physical activity lead to a decreased risk of AD. Mild to moderate amount of exercise decreases neuroinflammation by reducing the amount of intracellular neurofibrillary tangles and extracellular amyloid-beta plaques. Moderate and high intensities have demonstrated neuroprotective effects through the production of antioxidant enzymes and growth factors such as superoxide dismutase, e-NOS, BDNF, nerve growth factors, insulin-like growth factors and vascular endothelial growth factor and by reducing the production of ROS. Various animal model studies and clinical trials in elderly and middle age people are of the view that leisure activities and physical exercise not only slow the cognitive decline but improves cognitive components such as sustained attention, visual memory and frontal cognitive function in patients with mild to severe AD. Many more trials should be done to strengthen this association. 
